# Green method of stemming the tide of invasive marine and freshwater organisms by natural filtration of shipping ballast water

**DOI:** 10.1007/s11356-020-10839-4

**Published:** 2020-09-21

**Authors:** Jebarathnam Prince Prakash Jeba Kumar, Shunmugavel Ragumaran, Ganesan Nandagopal, Vijaya Ravichandran, Ramana Murthy Mallavarapu, Thomas M. Missimer

**Affiliations:** 1 Coastal Environmental Engineering Division, National Institute of Ocean Technology, Ministry of Earth Sciences, Govt. of India, Pallikaranai, Chennai, 600100 India; 2 National Center for Coastal Research, Ministry of Earth Sciences, Govt. of India, NIOT Campus, Chennai, India; 3 U. A. Whitaker College of Engineering, Emergent Technologies Institute, Florida Gulf Coast University, 16301 Innovation Lane, Fort Myers, FL 33913 USA

**Keywords:** Ballast water, Invasive species, Environmental impacts, Subsurface intake systems

## Abstract

Marine and freshwater pollution caused by transport of invasive species in shipping ballast water is a major global problem and will increase in magnitude as shipping of commodities increases in the future. An economical method to preclude biological organisms in the seawater used for ballast is to exclude them at the source port. Integrated natural filtration using onshore wells or seabed gallery systems has been thoroughly investigated for use as pretreatment for seawater desalination systems and has proven to be environmentally acceptable and economic. Thus, the use of this proven filtration technology to another issue, ballast water treatment, is an innovative method of providing marine organism free seawater by non-destructive means in port-based facilities. This method is ecosystem-friendly in that no chemicals or destructive processes are used. Design and construction of well or seabed gallery intake systems for production of ballast seawater are feasible in virtually all global port facilities.

## Introduction

Seawater used for ballast typically contains numerous macroscopic and microscopic organisms, which are discharged into the oceans worldwide. Thus, ballast water disposal is believed to be a primary vector for the spread of aquatic invasive species globally (Carlton 1985, 1999; Endresen et al. 2004; Takahashi et al. 2008; Tsolaki and Diamadopoulos 2010; Seebens et al. 2016; Carney et al. 2017; Seebens et al. 2017). Marine ship traffic is a critical part of the global economy by providing international delivery of goods and commodities. Each year between 3 and 5 billion metric tons of seawater are utilized as ballast water in shipping (Tsolaki and Diamadopoulos 2010). Global maritime traffic has been projected to increase 20-fold by 2050 to account for 80% of world trade, which could lead to a sharp rise in invasive species by 90% around the world if not controlled (Sardain et al. 2019).

The magnitude and diversity of marine organisms delivered in ballast water throughout the world include about 10,000 species transported between different biogeographic regions (Carlton 1999; Hewitt et al. 2009). Historical study of ship ballast water (freshwater) entering the North American Great Lakes revealed an average of 17 active species with varying densities of 10,000 to 8 billion individuals per vessel (Howarth 1981). Records of ballast water-mediated introductions and their threat to marine biodiversity, coastal economies, local cultures and livelihoods, and human health are well-documented by various studies (Sardain et al. 2019; Shimokawa 2012; Anil et al. 2002). Since the ballast water–transported species are alien to the new environment, they often cause harmful effects to the native biological community, thereby impacting economic and sociological conditions and establishing themselves as invasive species (ISs). In addition, there are also documented health effects based on the transport of disease-causing microorganisms, such as *Vibrio cholerae* (Takahashi et al. 2008).

The multiple impacts of ISs are recognized as major environmental threats to the marine and certain freshwater environments, causing predation and competition for food with native organisms and eutrophication in areas where discharge of ballast water containing dead organisms occurs (Halpern et al. 2008). Since the adoption of the Convention on Biological Diversity Strategic Plan for 2011–2020 by the International Union for Conservation of Nature (IUCN), there is traction on trying to achieve the realistic Aichi Target 9 of identifying invasive species and their pathways into non-native environments. Priority invasive species in ballast water need to be controlled or eradicated, and measures must be implemented to manage pathways to prevent their introduction (Castro et al. 2018; Krishnamurthy et al. 2018; Mapari et al. 2018). Therefore, establishment of effective and economic technological methods to limit the invasive species menace is necessary.

The purpose of this research is to evaluate the use of subsurface intake systems as a source of filtered seawater or freshwater to eliminate the marco- and microorganisms from ballast water at ports where it is uploaded. This natural filtration technology using wells or galleries has been demonstrated to be cost-effective in the improvement of water quality (pretreatment) in seawater reverse osmosis desalination facilities. The use of well-developed engineering applications in one field to another problem is considered to be an innovation, and thus, the focus of this research is the application of this well-developed technology to treatment of ballast water.

## Strategies for control of ballast water biological contamination control

There are three fundamental strategies that can be implemented to control the presence of invasive species in ship ballast water. First, the control measures can be implemented at the source where ballast seawater is pumped into the ship. Second, the ballast water can be treated within the ship before being discharged to the environment. Third, a combination of providing “clean” ballast at the source where it is pumped into the ship with later treatment to assure that no macroscopic species, ichthyoplankton, bacteria, or viruses can enter the environment during discharge.

### Physical and chemical treatment methods (destructive)

The International Maritime Organization (IMO) is the United Nations (UN) specialist agency that is responsible for the safety and security of shipping and prevention of marine and atmospheric pollution by ships. It sets the standards for maritime transport that promulgates recommended control measures included in the International Convention for the Control and Management of Ships’ Ballast Water and Sediments (IMO 2004). Because shipping is genuinely an international activity, the IMO sets technical standards and requirements regarding the regulatory control and management of ship ballast water and invasive species which are adopted by member states of the UN (IMO 2008a; IMO 2008b; IMO 2012). Many techniques that satisfy the IMO criteria are used to minimize or prevent the introduction of non-indigenous species into ballast water and to remove invasive species before discharge but be approved by the GESAMP-BWWG (IMO 2008b; IMO 2012; Tsolaki and Diamadopoulos 2010). The binding agreement established in 2004 mandated two management standards that should be applied as a choice of ballast water management. These standards are (1) standard D-1 on ballast water exchange that requires vessels to exchange their ballast water uploaded in coastal areas for ballast water from the open ocean “whenever possible 200 nautical miles from the nearest land and in the water at least 200 m in depth (Regulation B-4)” and (2) standard D-2 on ballast water performance that establishes water quality standards for ballast water treatment systems (BWTSs) (Gerhard et al. 2019).

Coarse filtration techniques are commonly used as an environmentally friendly method for ballast water treatment. Many multilevel filtration techniques do not have a significant effect on reducing microscopic planktonic organism concentrations in seawater pumped into the ship after debarking of cargo (Cangelosi et al. 2001; Cangelosi et al. 2014). The US Coast Guard does not specifically require some type of filtration in the treatment process but is commonly used in many treatment processes. Modern technology based, large-scale seawater filtration systems, like traveling water screens, used in coastal power plant intakes can effectively filter floatable debris and fish to reduce impingement and entrainment, but these coarse screens fail to screen ichthyoplankton, which consists of microscopic plankton, eggs, and larva of various fishes, prawns, and benthic organisms (Alimah and Parapak 2008; Jebakumar et al. 2018). Other techniques like mechanical separation and treatment include the use of ultraviolet radiation, heat treatmen, electric pulse applications, and chemical treatment were also adopted (Anil et al. 2002; Endresen et al. 2004; Tsolaki and Diamadopoulos 2010). These destructive methods eliminate most of the planktonic forms along with potential invasive species but leave behind contaminated seawater containing biodebris and changes of some dissolved organic carbon into assimilable organic carbon. In addition, excess oxidants and their chemical byproducts may be discharged into the marine environment, and larger debris generated would require disposal at a landfill or would have to be incinerated. Scientific documentation of invasive species worldwide indicates that insufficient mitigation efforts are being made to curtail spread of invasive species (Seebens et al. 2017). Hence, ballasting with seawater devoid of significant concentrations of life forms from the port is essential to minimize impacts from ISs.

### Use of natural subsurface filtration systems at ballast water intake locations

Over the past 40 years, seawater desalination has become an integral part of water supply strategies in many parts of the world, and the reverse osmosis process is the leading technology in terms of efficiency and cost (Ghaffour et al. 2013; Amy et al. 2017). Unfortunately, seawater contains an abundance of organic materials and compounds that collectively cause biofouling of the primary membranes, despite extensive pretreatment of the raw water (Flemming 1997; Vrouwenvelder et al. 1998). In recent years, considerable research has been conducted on the use of subsurface intakes to remove organic macroscopic debris, algae, and bacteria. These natural filtration–based intakes also remove significant parts of the smaller-sized organic matter, including transparent exopolymer particles and the biopolymer fraction of natural organic matter (Missimer 2009; Missimer et al. 2013; Rachman et al. 2014; Dehwah et al. 2015; Dehwah and Missimer 2016; Dehwah and Missimer 2017). This technology uses either shallow wells located adjacent to the shoreline or some type of gallery intake and has been used successfully to remove organic materials from the raw seawater that allows seawater reverse osmosis desalination plants to operate more economically with addition of chemicals (e.g., chlorine) and less cleaning of the membranes (Missimer et al. 2015). This technology could also be used to pretreatment ship’s ballast to remove invasive species.

### Use of combined subsurface filtration for ballast source with treatment before discharge

Subsurface intake systems remove all of the macroscopic forms of carbon but only some percentage of the bacteria (Dehwah and Missimer 2016, 2017). The bacteria that are not removed may be only the very small-sized genera known as ultramicrobacteria (Cavicchioli and Ostrowski 2003). Therefore, if potential pathogens are suspected of occupying the source ballast water, then disinfection of the water could be performed prior to final discharge only if necessary. The need to disinfect the ballast water could be eliminated by monitoring of the subsurface source water bacteria prior to using it for ballast. If no pathogenic bacteria or viruses are found, then there would be no reason to disinfect the source water. If deep water well intakes are used, it would be highly unlikely that the source water would contain any pathogens.

## Assessment of the technical feasibility and effectiveness of subsurface filtration for ballast water treatment

There are two aspects concerning the feasible use of subsurface intake technology to obtain “clean” ballast water. First, the issue concerning whether the geology near a port facility will allow the development of a well system or some type of gallery intake system. The presence of some type of aquifer is necessary for well development with yields sufficient to meet the ballast water demands. If a productive aquifer is not located near the port facility or the aquifer transmissivity is too low, then a seabed gallery or beach galley could be constructed instead of wells. Second, the intake type developed must provide a reliable supply of seawater that will effectively prevent the movement of invasive biological forms from the source water to the discharge location.

### Well intakes where the geology is favorable

**Fig. 1:**
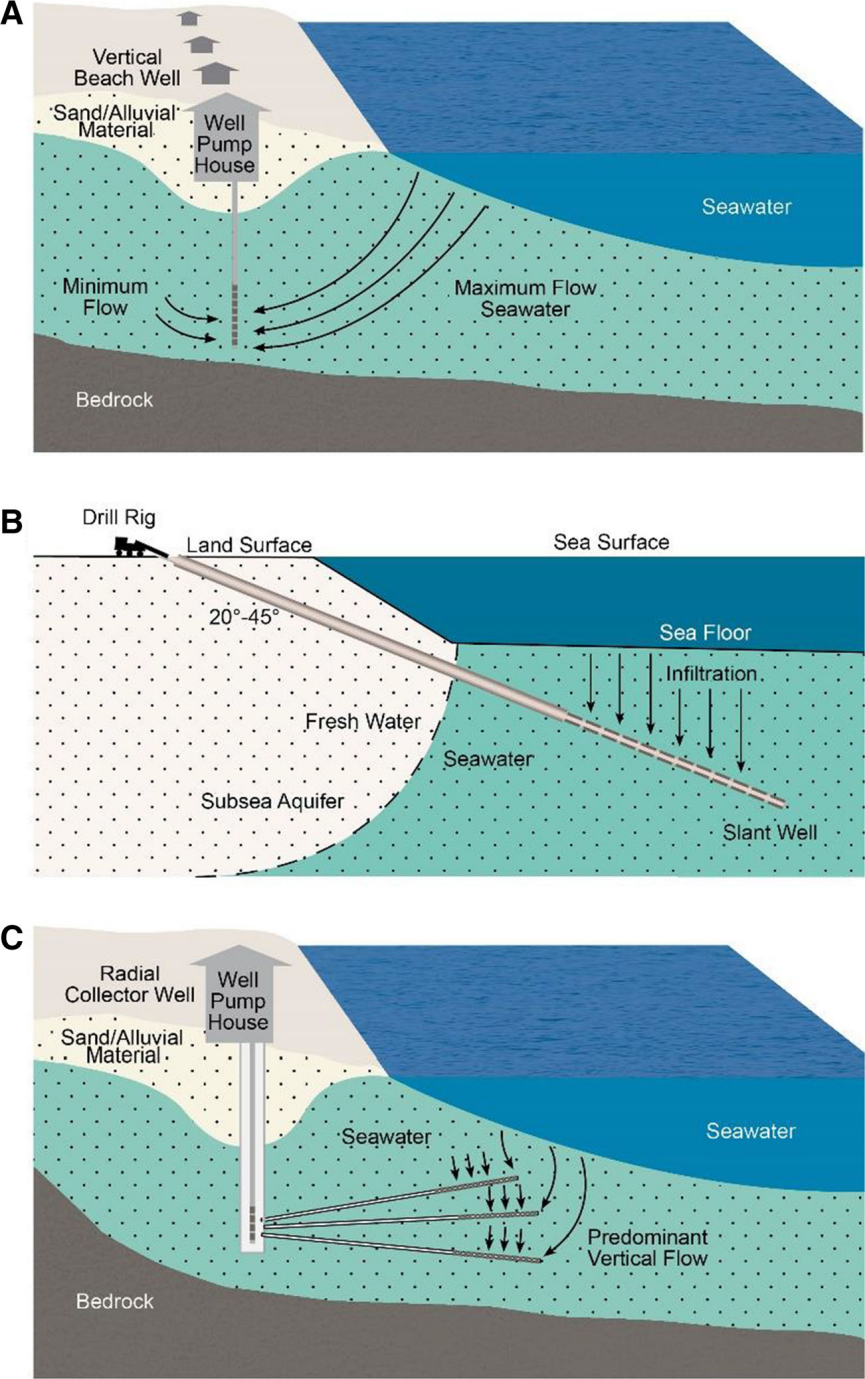
Well, intake designs for obtaining ballast water. **a** Conventional vertical well located near tidal water (or a beach). **b** Angle well that can be constructed at some distance from the shoreline. **c** Ranney well that would need to be constructed on the beach

**Fig. 2:**
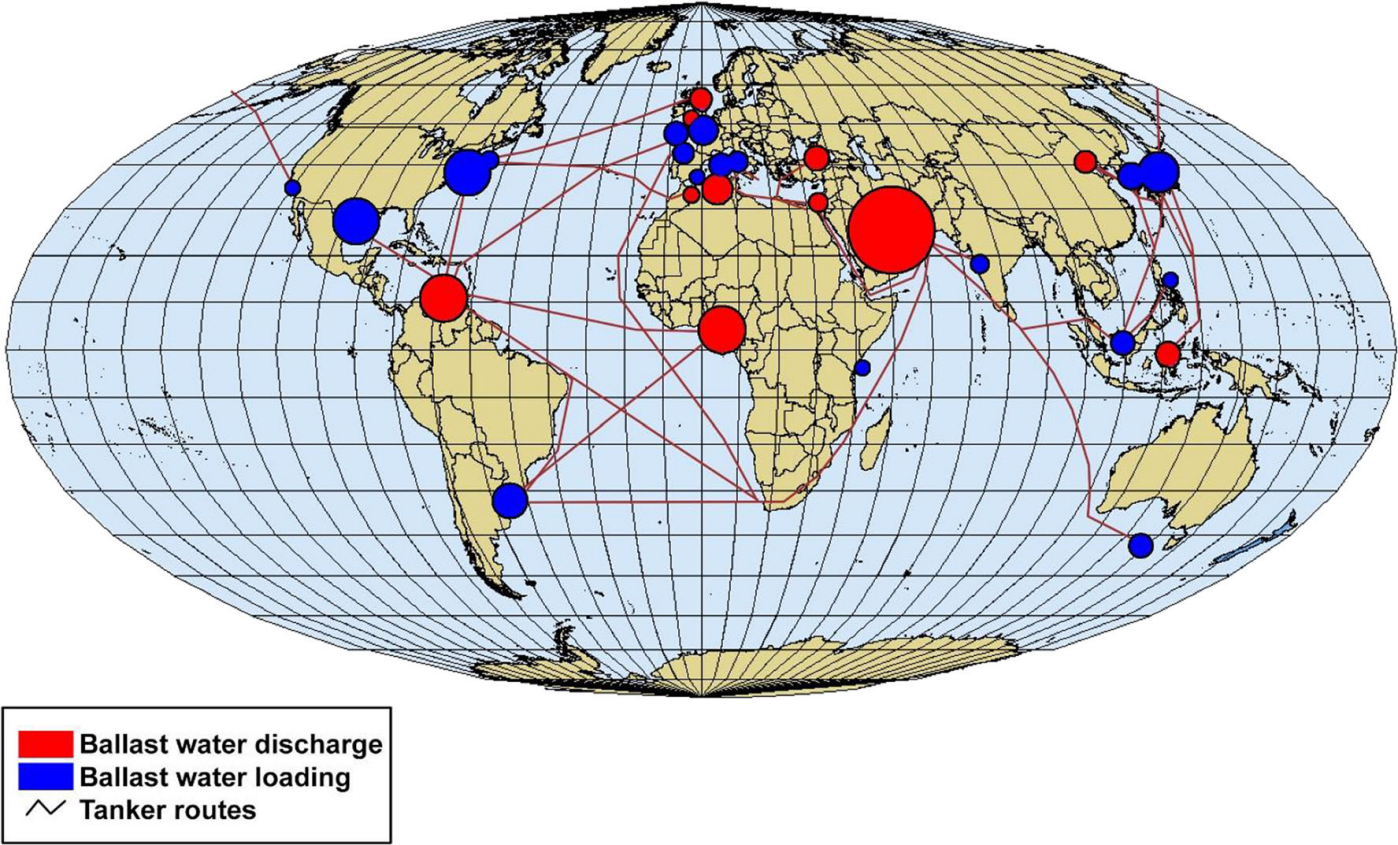
Locations of oil tanker shipping ports where ballast water is loaded and discharged (modified from Endresen et al. 2004)

A number of different well designs are available for use where there are permeable sediments at shallow deeps near a port location. Three designs are shown in Fig. 1, with the most common one being the conventional vertical well (Fig. 1a). A slant well can be used, as shown in Fig. 2b, but this design necessitates the use of specialized well drilling equipment that may not be present in some areas of the world. However, this design allows the well to be constructed at some distance from tidal seawater. Another well type is the Ranney well which also is a specialized type of well but can yield large quantities of seawater up to 50,000 m^3^/day/well (Fig. 1c) (Missimer et al. 2013). Two key issues in the design of these intake wells are that they must be hydraulically connected to the sea and are located away from any sources of groundwater contamination that could provide water quality issues at the point of ballast water discharge. Detailed design methods for well systems located near the shoreline are described in Missimer (2009), Maliva and Missimer (2015), and Williams (2015).

A review of major port locations where large oil tanker ships take on ballast water is provided by Endresen et al. (2004) (Fig. 2). Based on these shipping port locations, most of them have acceptable geology nearby that would allow successful development of well intake systems.

Measurements of the organic matter transport in well intake systems have been conducted at several locations around the world (Missimer 2009; Missimer et al. 2013; Rachman et al. 2014; Dehwah et al. 2015; Dehwah et al. 2016; Dehwah and Missimer 2016; Dehwah and Missimer 2017). Select data on the removal of combined algae and cyanobacteria and marine bacteria from these investigations are summarized in Table 1, and additional data are provided in the reference papers. In most cases, well intakes remove 100% of the algae (and ichthyoplankton) from the seawater when comparing the concentration in the raw seawater to that measured in the well discharge. Since the cyanobacteria are relatively small in size compared with the major algae, they can be used as a proxy for other pathogens of similar size in freshwater systems. There is also effective removal of marine bacteria with an efficiency range from 84.0 to 99.8%. The removal percentage increases with the seawater flow path length from the seabed to the well locations (Dehwah et al. 2016; Dehwah and Missimer 2016). Therefore, well intake systems are quite effective in the removal of biological organisms as small as marine bacteria. It is believed that the bacteria that pass through well filtration (and reverse osmosis membranes) are the ultramicrobacteria, which have a cell volume of < 0.1 μm^3^. (Cavicchioli and Ostrowski 2003). These oligotrophic marine bacteria constitute a large percentage of the bacteria in the sea. In addition, they may be resistant to chlorination. The sampling and analytical methods used in the referenced investigations are included in the cited references.

**Table 1:** Representative effectiveness of combined algae and cyanobacteria and marine bacteria removal by well intake systems (^1^Dehwah and Missimer 2016; ^2^Dehwah et al. 2016; ^3^Rachman et al. 2014)

Location	Algae		Bacteria	
	Original number	Percent removed	Original number	Percent removed
^1^North Obhor, S.A.	Number/mL	(%)	Cfu/mL	(%)
Well 1	129,738	100	520,350	98.9
Well 2	129,738	100	520,350	97.5
Well 3	129,738	100	520,350	98.4
Well 4	129,738	100	520,350	97.9
^1^Coniche, Jeddah, S.A.				
Well 1	89,033	100	254,450	91.8
Well 2	89.033	100	254,450	90.3
Well 3	89,033	100	254,450	90.0
Well 4	89,033	100	254,450	80.5
^1^South Jeddah, S.A.				
Well 1	49,923	98.3	216,400	94.5
Well 2	49,923	99.5	216,400	89.0
Well 3	49,923	98.4	216,400	84.4
Well 4	49,923	99.8	216,400	84.6
^2^North Obhor, S.A. #2				
14 wells	129,738	100	520,350-1,356,600	97 avg.
^3^Sur, Oman				
Well SR1b	194,310	100	702,609	99.3
Well SR2b	194,310	100	702,609	99.8
Well SR3b	194,310	100	702,609	99.6
Well SR4b	194,310	100	702,609	99.3
Well SR5b	194,310	100	702,609	99.6

### Engineering and cost aspects of well filtration systems

There is an extensive amount of literature on use of wells to provide filtered water to desalination plants, some of which have high capacity. The largest capacity using wells for a seawater reverse osmosis water treatment plant is currently about 160,000 m^3^/d at Sur Oman, and the largest capacity seawater gallery has a yield of 103,000 m^3^/d that is located at Fukuoka, Japan (Missimer et al. 2013). However, it is not difficult to build these facilities at much higher capacity.

The estimated capital cost for the construction of treatment facilities using well filtration can be estimated based on existing desalination plant costs. A series of cost curves for the investment costs for SWRO desalination plants were developed by Ghaffour et al. (2013), which included intake costs. Based on a wellfield capacity of 100,000 m^3^/d capacity, the investment cost would range between $50 and $200/m^3^. This is directly applicable to systems used to develop ballast water and is based on the aquifer characteristics at the specific port site. Also, as the capacity increase, the cost tends to decline.

Because of the small size of the bacteria discharged from seawater wells, the removal of target classes of marine organisms of concern in ballast water is achieved. Based on the range of sizes of the groups considered within the D-2 standard under the IMO (2012) standards, all of these classes would be removed with no pathogenic bacteria remaining in the filtered water (Table 2). The size of microorganisms being discharged from well systems is commonly under 1 μm in diameter and likely occurs in the ultramicrobacteria size of 0.02 to 0.1 μm range.

**Table 2:** IMO/USCG ballast water performance standard D2 sets the limits of active organisms as shown

Microorganism category	Control limit
Viable/living organisms, size > 50 μm	< 10 viable/living cells/m^3^
Viable/living cells, size 10–50 μm	< 10 viable/living cells/mL
Toxicogenic *Vibrio cholerae*	< 1 colony-forming unit/100 mL
*Escherichia coli*	< 250 colony-forming units/100 mL
Intestinal enterococci	< 100 colony-forming units/100 mL

In addition, the organic chemistry of the discharge water from wells and seabed galleries is lower than the IMO standard D2 (Table 3).

**Table 3:** Suspended solids and organic concentration standard under IMO/USCG ballast water performance standard D2

	Salinity		
	Marine 28–36 PSU	Brackish 10–20 PSU	Fresh < 1 PSU
Dissolved organic carbon (DOC)	> 1 mg/L	> 5 mg/L	> 5 mg/L
Particulate organic carbon (POC)	> 1 mg/L	> 5 mg/L	> 5 mg/L
Total suspended solids (TSS)	> 1 mg/L	> 50 mg/L	> 50 mg/L

**Fig. 3:**
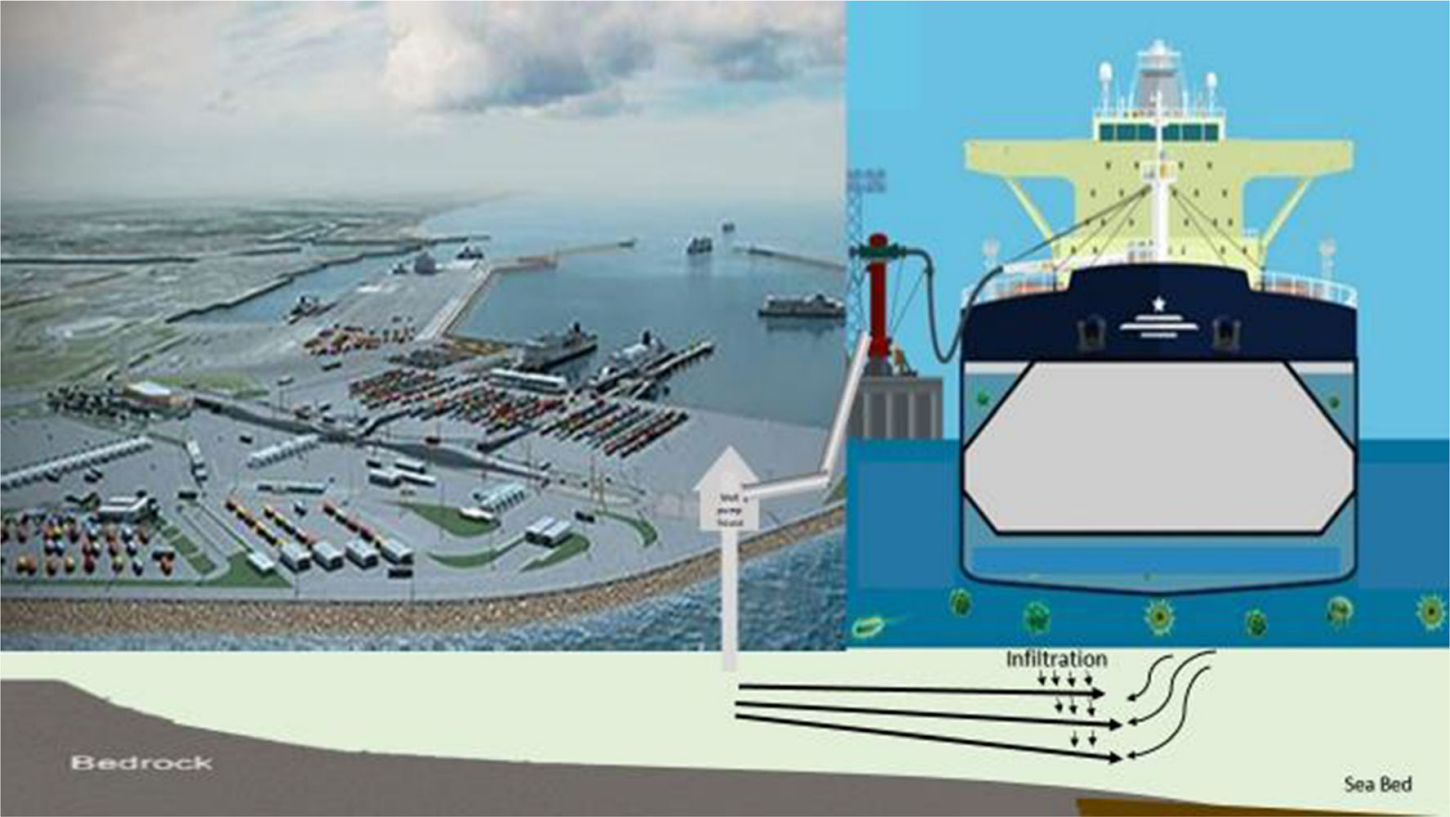
Schematic diagram of the use of well filtration systems on seabed infiltration galleries to supply ship ballast water. Note that pipelines and storage tanks would be installed adjacent to the docking facilities

The filtered water could be delivered to the ships with installed infrastructure at the port or could be conveyed under the seabed via pipelines to anchorage points. A design concept for the delivery of the filtered water is shown in Fig. 3.

Well filtration systems are environmental friendly in that they occur in locations near beaches and can be installed in a manner to make them part of the onshore coastal infrastructure. For example, numerous water supply wells are used at many resort island environments without causing environmental impacts (Missimer et al. 2013; Rachman et al. 2014). In addition, well systems occurring immediately adjacent to tidal water cannot cause saltwater intrusion into freshwater aquifers because no freshwater occurs between tidal water and the wells. Two water volume scenarios have been suggested for port treatment facilities which are (1) a treatment capacity of 2000 m^3^/h, onsite storage of 25,000 m^3^, and a residence time of 24 h and (2) 20,000 m^3^/h, onsite storage of 25,000 m^3^, and a residence time of 24 h (National Academies Press 1996).

These installed capacities can be met on the low side by a well system based on existing systems used by SWRO desalination intakes (Sur, Oman well system, installed capacity of 160,000 m^3^/d) and on the high side by a seabed gallery system which has essentially unlimited capacity (Dehwah and Missimer 2017).

### Seabed gallery intakes where the geology for well intakes is unfavorable

**Fig. 4:**
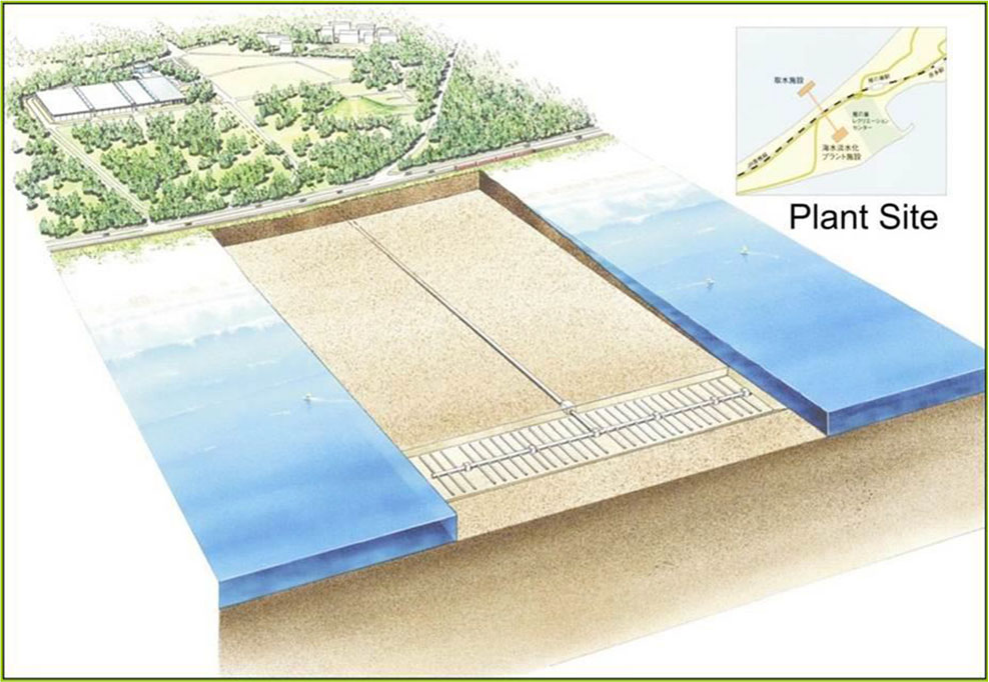
Seabed filter design of the 103,000 m^3^/day seabed gallery system used as a seawater intake for the Fukuoka, Japan desalination plant (from Hanamo et al. 2006). The system has been operating for more than a decade with no issues such as clogging

In some port locations, the geology of the adjacent shoreline or beneath the port may be unfavorable for the successful development of a well intake system. In these areas, a seabed gallery could be developed to obtain seawater free of algae and a high percentage of marine bacteria. Since the flow pathway through a seabed gallery system is generally less than a well system, the removal of bacteria is not as effective in terms of overall percentage (Dehwah and Missimer 2017). Experimental work conducted by Dehwah and Missimer (2017) verified that 100% of the algae are removed in the filter and up to 84% of the marine bacteria are removed. They also found that the initial removal percentage of bacteria could be as low as 50%, but increased as the filter matured, which could take up to several months of operation. This finding was similar to that found in a large-scale operating seabed filter in Fukouka, Japan, where the silt density index of the filtered seawater improved significantly over 12 years of operation (Fig. 4; Hanamo et al. 2006; Shimokawa 2012). The design criteria for seabed gallery systems are discussed in detail by Missimer et al. (2015). This type of intake can be constructed near the shoreline or offshore depending on localized conditions, such as the sedimentation rate.

## Discussion

In science and engineering, technology that has been developed and applied for another purpose can be used to solve other environmental or engineering problems which is considered to be an advancement and an innovation. An example of this concept is the development of advanced oxidation technology to treat industrial wastewater for reuse in manufacturing facilities (Rojas et al. 2010). The process facilitated reuse of water and caused a reduction in the overall water consumption. This technology has been applied in recent years to the removal of trace concentrations of emerging contaminants (Tufail et al. 2020).

Natural filtration technology using wells or seabed gallery systems was successfully developed and tested for pretreatment of seawater in the desalination process. This same technology can be applied to filter seawater through wells (aquifer treatment), or seabed galleys can be used to provide high-quality seawater to be used as ballast in ships at most port facilities. This filtered water is essentially free from harmful marine or freshwater organisms. There are, however, some infrastructure issues at large ports that must be considered in design of ballast water supply systems. The engineering design will require an initial assessment of the water volumes required and the support water storage and pipelines required for delivery of water to the ships. Individual well yields are based on site-specific aquifer hydraulic properties, and the number of wells and their location would have to be coordinated with the other infrastructure. Some port locations (e.g., Port of Miami) would have no issue with development of very high yield wells which could preclude the need for tank storage, because of the very high transmissivity of the underlying Biscayne aquifer.

**Fig. 5:**
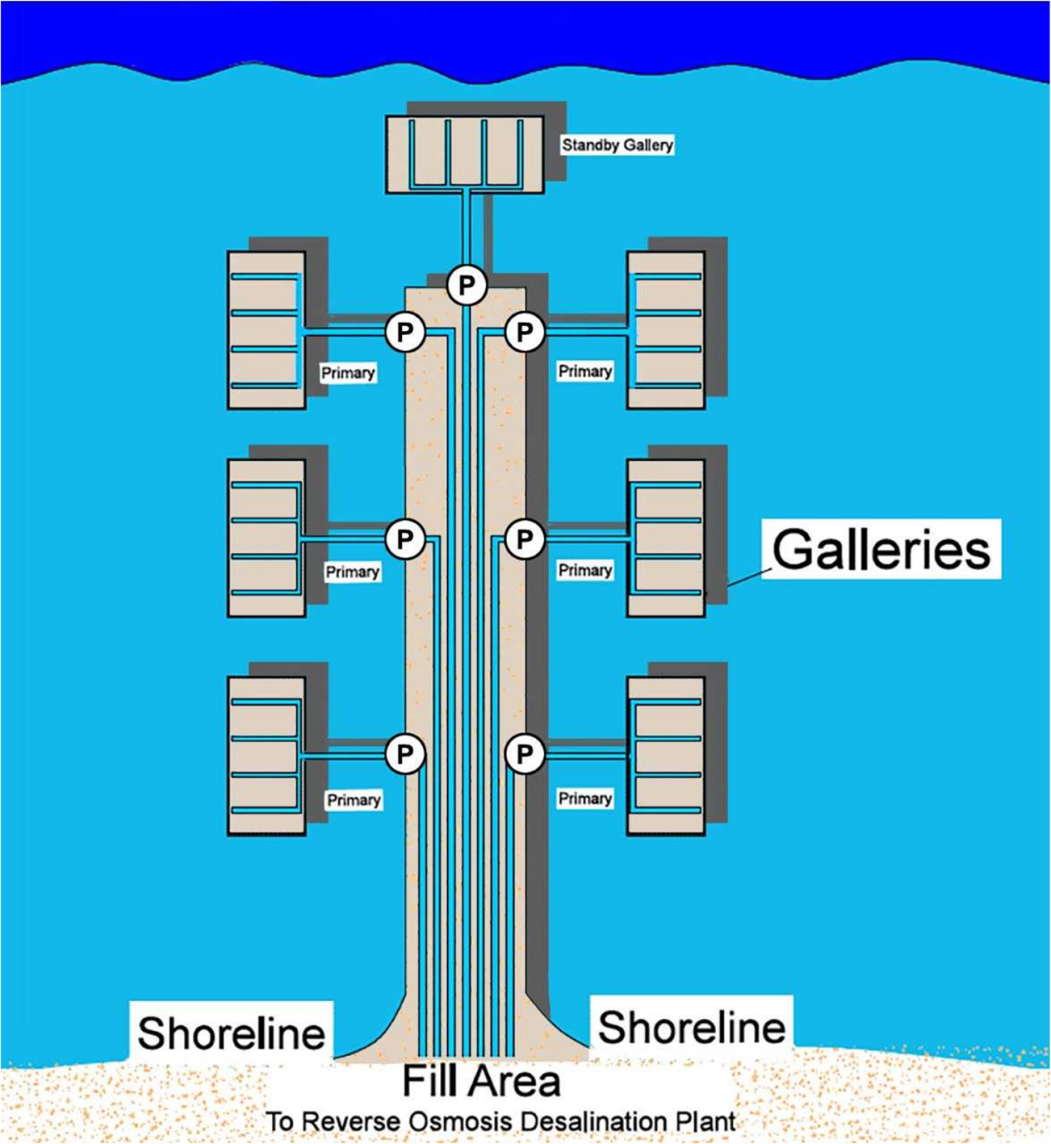
Example of a seaport gallery system that could be installed adjacent to the docks of a port facility (from Missimer et al. 2015)

There will be locations where well yields are insufficient to meet the needs of the overall ballast water requirements. At these locations, a seabed filter system would be the best design solution because systems can be designed to meet any potential water volume required (Missimer et al. 2015). An example of a very large-scale seabed gallery system is shown in Fig. 5. This was initially designed to supply a high-capacity seawater reverse osmosis desalination plant, but could be easily modified to operate in a port facility, perhaps directly adjacent to docks and beneath ships. The top of the filters would have to be periodically cleaned using a mini dredge to remove 10 to 20 cm of sediment. The cleaning time would be dependent on the turbidity of the water at the facility. This issue would be evaluated during the design of the system.

As shown in the data from operating well intake systems, all of the algae (including marcoscopic ichthyoplankton) are removed in the filtration process. However, not all of the marine bacteria are removed, which may not be problematical because most of them are very small and not pathogenic. If this is an issue, some chlorination could be used in the storage tanks at the ports to remove any remaining bacteria. Proper environmental investigations should be conducted before disinfection is considered.

## Conclusions

Transport of invasive species in ballast water (3 to 5 billion tons per year) used in shipping from one location to another has caused considerable environmental harm over the past century. It is proposed that the seawater pumped onto ships as ballast should be pretreated using a natural subsurface filtration process, a technology initially developed to improve seawater desalination plant performance. Two environmentally friendly filtration methods, wells or galleries, can be used to provide “clean” seawater, devoid of both macroscopic (algae and ichthyoplankton) and microscopic living marine organisms. Both methods provide the necessary pretreatment without the use of chemicals (i.e., chlorine) and/or expensive post-treatment of the ballast water before discharge. This technology has been thoroghly researched in desalination applications in terms of effectiveness for removal of particulate matter, algae, and bacteria. It has direct application to balast water treatment.
